# Statistical shape modelling of the thoracic spine for the development of pedicle screw insertion guides

**DOI:** 10.1007/s10237-022-01636-8

**Published:** 2022-09-19

**Authors:** Gordon Wai, Wan Rusli, Shaaz Ghouse, David C. Kieser, Angela Kedgley, Nicolas Newell

**Affiliations:** 1 Department of Mechanical Engineering, Imperial College London, London, UK; 2 Department of Bioengineering, Imperial College London, White City Campus, London, W12 7TA UK; 3 Department of Orthopaedics and Musculoskeletal Medicine, Christchurch School of Medicine, University of Otago, Christchurch, New Zealand

**Keywords:** Spine, Statistical shape modelling, Surgical guides, Pedicle screws

## Abstract

Spinal fixation and fusion are surgical procedures undertaken to restore stability in the spine and restrict painful or degenerative motion. Malpositioning of pedicle screws during these procedures can result in major neurological and vascular damage. Patient-specific surgical guides offer clear benefits, reducing malposition rates by up to 25%. However, they suffer from long lead times and the manufacturing process is dependent on third-party specialists. The development of a standard set of surgical guides may eliminate the issues with the manufacturing process. To evaluate the feasibility of this option, a statistical shape model (SSM) was created and used to analyse the morphological variations of the T4–T6 vertebrae in a population of 90 specimens from the Visible Korean Human dataset (50 females and 40 males). The first three principal components, representing 39.7% of the variance within the population, were analysed. The model showed high variability in the transverse process (~ 4 mm) and spinous process (~ 4 mm) and relatively low variation (< 1 mm) in the vertebral lamina. For a Korean population, a standardised set of surgical guides would likely need to align with the lamina where the variance in the population is lower. It is recommended that this standard set of surgical guides should accommodate pedicle screw diameters of 3.5–6 mm and transverse pedicle screw angles of 3.5°–12.4°.

## Introduction

Pedicle screws are the most common technique for spinal fixation and fusion as they reliably anchor instruments to the strongest region of the vertebra (Deng et al. 2016). Historically, insertion has been performed freehand, relying on the accuracy of the surgeon. However, there is a substantial learning curve for accurate screw placement. Gonzalvo et al. (2009) found that experience of inserting at least 60 thoracic pedicle screws under the supervision of an experienced surgeon is needed before an apprentice can insert screws independently.

Malposition of pedicle screws dominates the literature investigating post-surgery complications, as it can lead to vascular and neurological damage (Du et al. 2016). Incidence rates of malposition range from 0 to 42% and appear to be dependent on the skills of the surgeon (Merloz et al. 1998; Şarlak et al. 2009). Furthermore, the incidence of complications related to malposition has been reported to be as high as 42% (Tang et al. 2014). Importantly, even if patients with malpositioned screws are asymptomatic, chronic irritation of surrounding anatomy may cause major complications to develop beyond the follow-up period of published studies (Hicks et al. 2010). Consequently, Di Silvestre et al. (2007) stated a 4.2% rate of reoperation on patients with malpositioned screws, despite most patients being asymptomatic. Pedicle screw positioning also affects the quality of fixation and fusion. Incorrectly positioned screws can lead to screw loosening (Li et al. 2010), reducing fixation and requiring revision surgery.

Advances in additive manufacturing techniques mean it is now possible to use intraoperative surgical guides to assist pedicle screw insertion, with one of the benefits being increased accuracy compared to the freehand technique (Wilcox et al. 2017). These guides are particularly useful in the thoracic spine, where pedicles are small and the margin for error is lower than other regions of the spine (Chen et al. 2016). 3D-printed pedicle screw guides have been reported to reduce the malposition rate of freehand insertion by 14%, with even larger improvements seen in the thoracic spine (25% reduction) (Cecchinato et al. 2019).

Significant reductions in operating time have also been reported, which is likely to reduce related complications such as infection and blood loss (Deng et al. 2016). Fluoroscopy exposure to both patients and surgeons is also reduced by up to four times (Guo et al. 2017). Cecchinato et al. (2019) have shown overall radiation dose to the patient when using surgical guides is lower even when accounting for the preoperative CT scan. This is important as there are concerns that intraoperative fluoroscopy may cause an increased risk of tumours in orthopaedic surgeons and patients (Bratschitsch et al. 2019).

These guides are also much cheaper than other pedicle screw navigation techniques, such as robotic-assisted or CT-based navigation (Renson et al. 2014; Menger et al. 2018). Still, the use of patient-specific guides can result in a lead time of over 3 weeks (Medacta International 2017). As a result, these guides cannot be used for urgent surgeries and may worsen patient waiting times.

A set of standardised surgical guides may remove the need of patient-specific guide production completely, whilst retaining their low cost. This requires a detailed understanding of the variations of the posterior elements of vertebrae. A statistical shape model (SSM) may provide valuable information regarding the anatomical variations of vertebrae within a population. The aim of this work was to create an SSM to identify areas of low and high anatomical variation within the population such that the design requirements of a standardised set of pedicle screw guides can be ascertained.

**Fig. 1:**
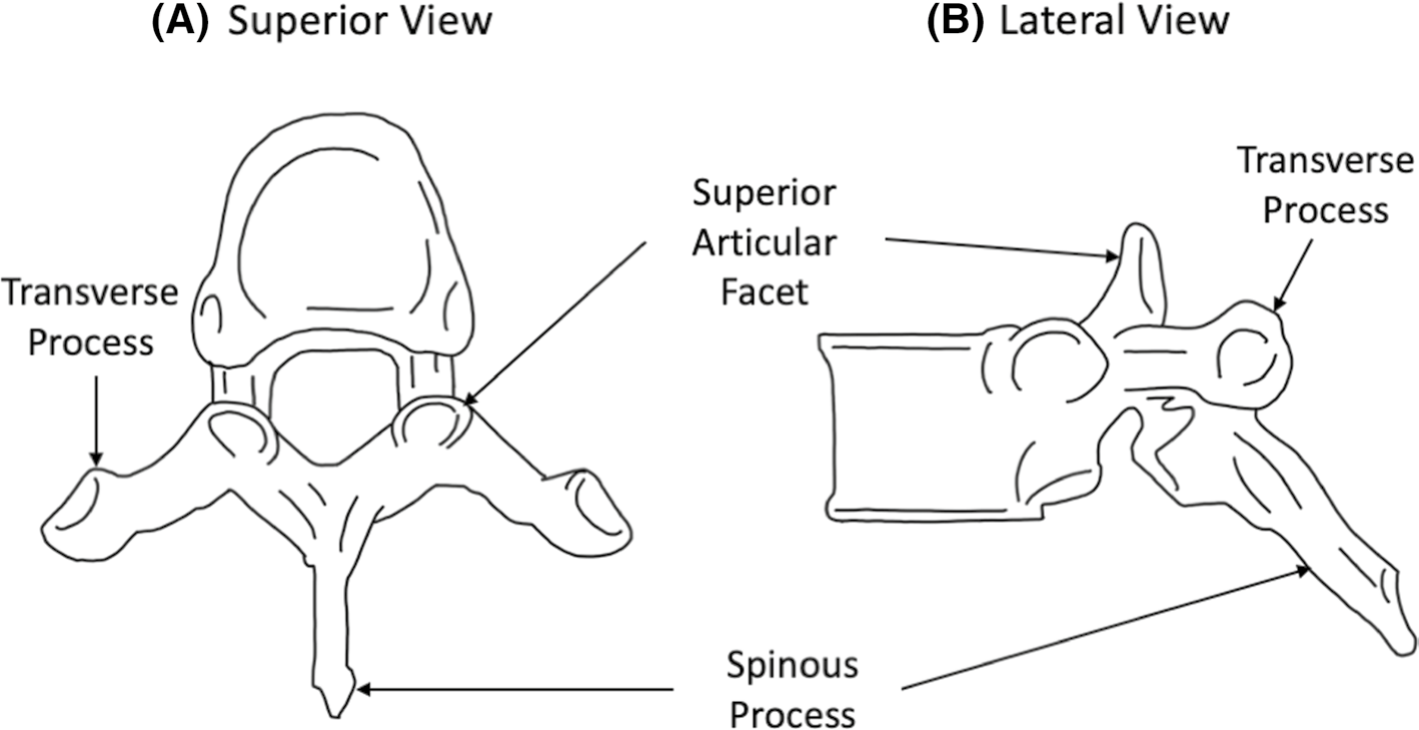
Diagrams of the four important vertebrae dimensions for surgical planning of pedicle screw insertion. Adapted from Bijendra et al. (2018) CC BY 4.0

This study focused on the T4–T6 portion of the thoracic spine where pedicles are smallest and navigation techniques have the greatest impact on positional accuracy (Cecchinato et al. 2019). Patient-specific surgical guides tend to anchor onto the posterior elements of the thoracic vertebrae (Fig. 1). Thus, analysis of the SSM results will also focus on the posterior elements of the vertebrae.

## Materials and methods

### Statistical shape modelling

Training data were obtained from the Visible Korean Human dataset (Park et al. 2006). Pre-segmented 3D models of the thoracic vertebrae (T4–T6) were checked for errors against the CT scans and corrected in 3D Slicer (version 4.10.2, https://www.slicer.org, accessed 01 November 2019) (Fedorov et al. 2012). The CT scans had an image spacing of 0.832 × 0.832 × 1.000 mm. Poor-quality CT scans, with ambiguous morphology, were omitted. This resulted in the original dataset of 112 patients (55 females and 57 males), reduced to a dataset of 90 patients (50 females and 40 males), with an age range of 28–60 years (mean 54.3, median 57.5). The heights of patients ranged from 146 to 176 cm (mean 160.4 cm, median 160 cm). The three vertebral levels, T4–T6, were pooled for analysis, resulting in 270 samples of one shape (90 patients × 3 vertebral levels from each patient).

Reasoning for pooling the levels was threefold. (1) It was desirable to develop a surgical guide/segmentation algorithm that could be used for all three levels of this region rather than one for each level. Therefore, it was decided that analysing the three levels as one shape rather than three would provide broader, more valuable insights for this application whilst also being simpler to analyse. (2) Anatomical differences between directly adjacent vertebrae are minor. Three-dimensional quantitative analysis by Panjabi et al. (1991) shows a gradual change in vertebrae dimensions when moving across the vertebral levels (< 10.5% change in endplate width/depth and less than 7.2% change in vertebral body height between T4 and T6 levels). Therefore, grouping the levels was unlikely to lead to major differences in results. Finally, (3) grouping the three levels had the added benefit of increasing the size of the training dataset used to create the SSM.

Modelling was performed using a statistical shape modelling pipeline, developed by Rusli and Kedgley (2019), consisting of four main steps: (1) Alignment (rigid registration) was performed using the rigid Coherent Point Drift (CPD) algorithm developed by Myronenko and Song (2010). CPD algorithms treat alignment as a probability density estimation problem, with one set being the datapoints and the other representing the Gaussian mixture model (GMM) centroids. The GMM centroids move “coherently” as a group to preserve the topological structure of the point sets. Though the algorithm that was used includes a scaling parameter, the pipeline allows this to be held constant. Scaling was not performed as it was recognised that this would affect how measurements of the SSM could be interpreted. (2) To enable the course to fine registration process, models were then sampled using a relevance-based sampling algorithm developed by Rodolà et al. (2015). The relevance-based sampling algorithm is dependent on the features of the region being sampled, such that it utilises the highest performing sampling algorithm for a given feature. (3) Non-rigid registration was carried out in two stages. Coarse non-rigid registration was performed using the non-rigid CPD algorithm developed by Myronenko and Song (2010). Fine non-rigid registration was then used to improve accuracy, using the local optimisation algorithm developed by Li et al. (2008), on the original samples, which removes the effect of the sub-sampling described in step 2. Finally, (4) projection-pursuit principal component analysis (PCA) was used for dimensionality reduction (Croux et al. 2007). Projection-pursuit PCA is suitable due to the high dimensionality of the problem. The number of principal components was limited to n-1, where n is the number of samples. Principal components representing more than 5% of the morphological variation were analysed further (Rusli and Kedgley 2019), as the variation in subsequent principal components was on the order of the voxel size of the CT scans. Leave-one-out analysis was performed to assess the generalisability of the model.

### Distance maps

The SSM was used to create 3D models of the mean shape of the population. 3D models of the variation along the principal components ± 3 standard deviations from the mean were also created (± 3 standard deviations from the mean were chosen as they are conventionally considered to include all values within a normally distributed population, i.e. 99.7% ≈ 100%). These models were overlaid with the mean shape used to create distance maps of the principal components’ variation from the mean.

### Pedicle screw trajectory measurements

**Fig. 2:**
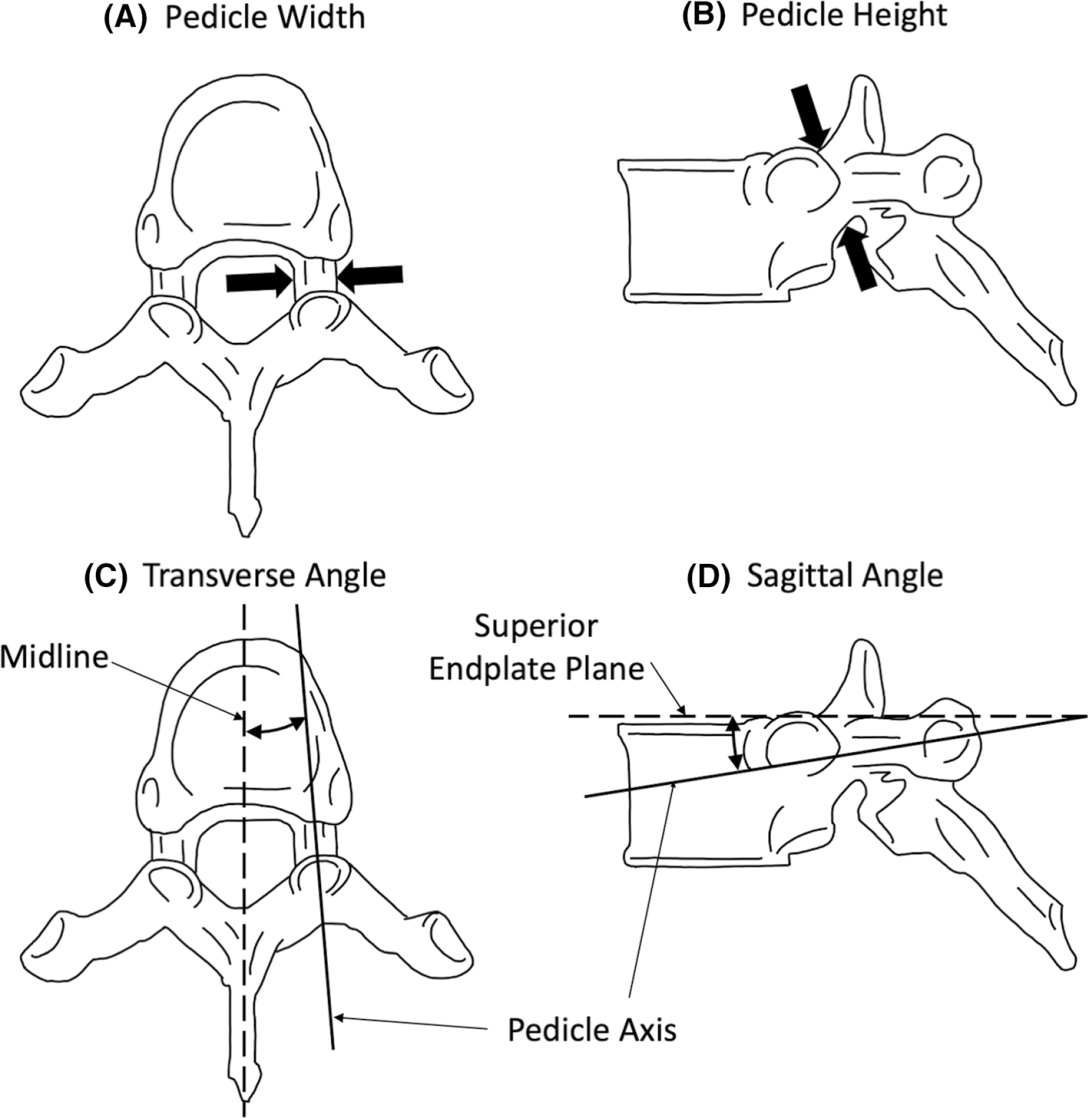
Diagrams of the four important vertebrae dimensions for surgical planning of pedicle screw insertion. Adapted from Bijendra et al. (2018) CC BY 4.0

There are four important dimensions of the vertebrae to be considered during surgical planning when using the straightforward and anatomical trajectory (Kuklo 2009; Oshina et al. 2018): pedicle width, pedicle height, transverse pedicle angle, and sagittal pedicle angle (Fig. 2).

**Fig. 3:**
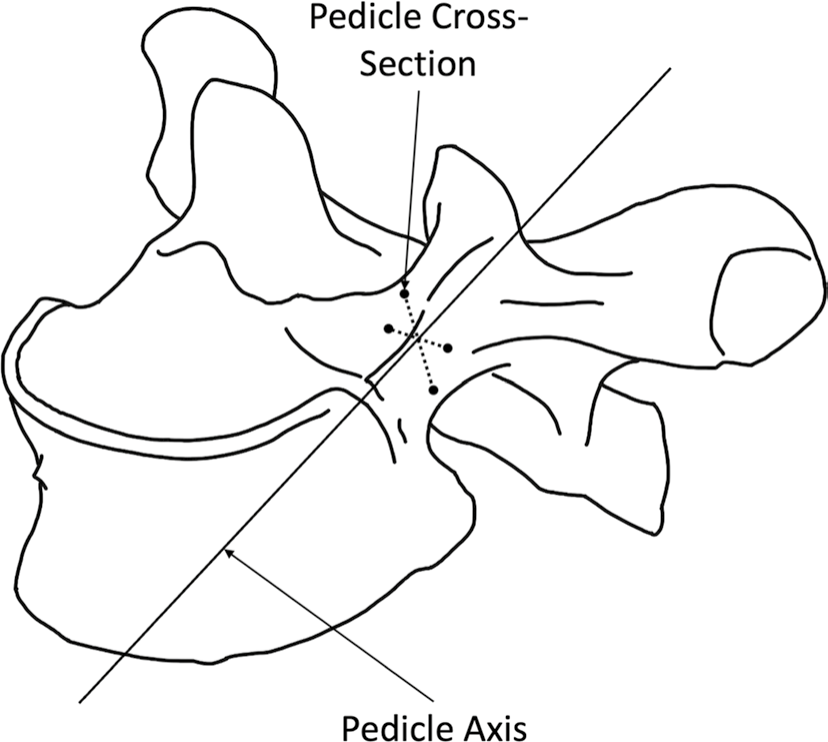
Definition of the pedicle axis. Black dots are the superior, inferior, lateral, and medial margins of the pedicle selected using the measurement tool. Adapted from Anatomy Standard (2020) CC BY 4.0

The generated models were used to take measurements of the key dimensions’ variations along the principal components in SolidWorks (v2020, Dassault Systèmes, SolidWorks Corp., Waltham, MA, USA). The pedicle width and height were taken using the SolidWorks measurement tool to select the superior, inferior, lateral, and medial margins of the pedicle. The cross section of the pedicle was then used to create a line showing the pedicle axis projection into the vertebrae (Fig. 3).

To find the transverse and sagittal angles, a plane parallel to the superior endplate was defined by selecting three points on the surface of the endplate. The midline was defined on this plane, from the most anterior point of the vertebral body to the most posterior point of the spinous process (Fig. 2C). The transverse angle was then taken as the angle between this line and the line of pedicle projection. The sagittal angle was the angle between the plane on the superior endplate and the line of pedicle projection (Fig. 2D). To account for measurement error, this process was repeated three times by the same investigator (GW). Each dimension was then averaged with the range of measurements recorded. Measurements were then compared against previous morphological studies.

## Results

**Fig. 4:**
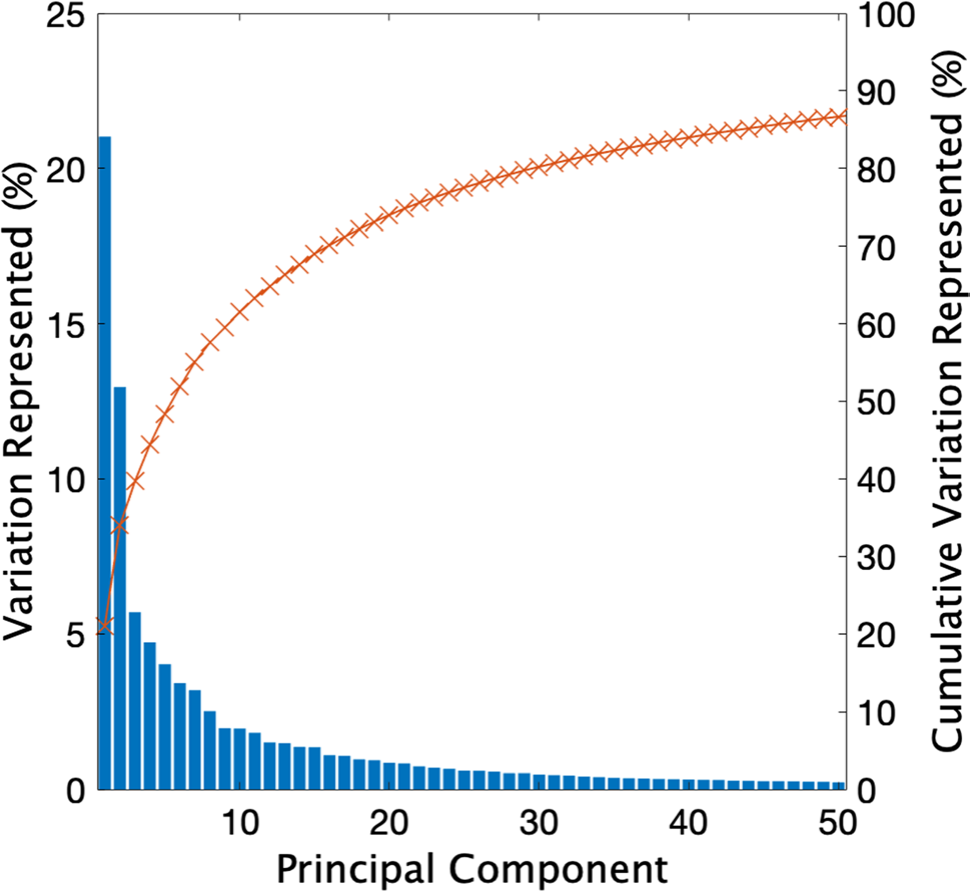
Separate (left *y*-axis) and cumulative (right *y*-axis) variation in morphology of the first 50 principal components with T4–T6 grouped together

This SSM comprised 269 principal components (PCs) representing the morphological variance of the T4–T6 vertebrae. The variance captured by the first 50 PCs is shown in Fig. 4. The remaining PCs were omitted as they represented very little variation (< 0.25%). The root mean squared error of the leave-one-out analysis was 0.24 mm. This was considerably less than the voxel size of the CT scans (0.832 × 0.832 × 1.000 mm) and was therefore considered acceptable.

**Fig. 5:**
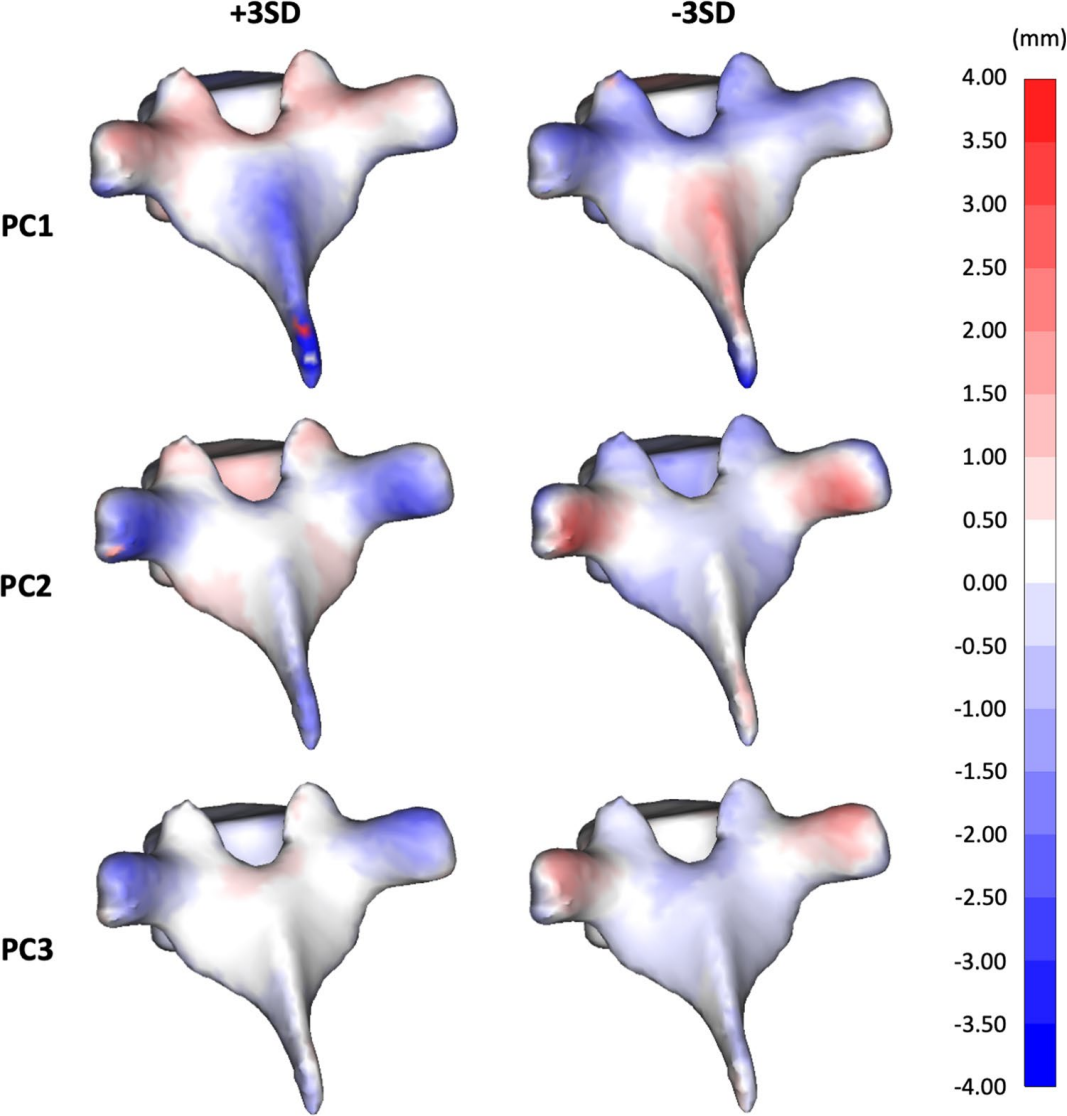
Distance maps representing the morphological variations in thoracic vertebrae (T4–T6) along the first three principal components − 3 standard deviations (SD) from the mean (left column) and + 3SD from the mean (right column)

The morphological variations in the thoracic vertebrae (T4–T6) along the first three PCs captured 39.7% of the variance within the population (Fig. 5). The first PC showed high variance along the superior aspect of the transverse process, superior articular process, and spinous process. In the second PC, high variation was seen in the inferior aspect of the transverse process. The third PC also showed high variance along the superior aspect of the transverse process, with less variance in the spinous process. The high variability (~ 4 mm) in the anatomy of the posterior aspects of the vertebrae means that the design of a standardised set of surgical guides is likely to be challenging.

The first and second PCs showed that within the population the transverse pedicle angle varies between 3.5° and 12.4° (Table 1). All three PCs showed variance in the pedicle width, with the third showing the largest range of 4.7–6.2 mm.

**Table 1:** Variation of pedicle dimensions of the first three principal components (ranges are measurement errors) ranging from + 3 standard deviations (SD) to -3SD

	+ 3SD	+ 2SD	+ 1SD	Mean	− 1SD	− 2SD	− 3SD
PC1							
Pedicle width (± 0.2 mm)	5.9	5.8	5.7	5.6	5.5	5.4	5.4
Pedicle height (± 0.2 mm)	11.0	10.9	10.7	10.5	10.5	10.5	10.3
Transverse pedicle angle (± 0.5°)	12.4	11.1	9.6	8.8	5.7	4.9	4.8
Sagittal pedicle angle (± 0.5°)	16.5	15.3	13.9	14.2	15.5	15.8	16.1
PC2							
Pedicle width (± 0.2 mm)	5.1	5.4	5.5	5.6	5.7	5.9	6.0
Pedicle height (± 0.2 mm)	9.4	9.7	10.1	10.5	10.8	11.3	11.5
Transverse pedicle angle (± 0.5°)	3.5	5.8	10.4	8.8	8.2	10.9	11.0
Sagittal pedicle angle (± 0.5°)	15.8	17.7	18.4	14.2	14.8	14.0	14.8
PC3							
Pedicle width (± 0.2 mm)	6.2	5.9	5.8	5.6	5.3	5.1	4.7
Pedicle height (± 0.2 mm)	11.5	11.4	10.8	10.5	10.0	9.2	9.0
Transverse pedicle angle (± 0.5°)	7.1	8.1	9.8	8.8	7.5	8.8	6.8
Sagittal pedicle angle (± 0.5°)	13.5	16.8	15.1	14.2	17.1	19.6	21.2

The pedicle dimensions in Table 1 were compared to previous studies that performed a morphological analysis on thoracic vertebrae (summarised in Table 2). A search for publications was performed for any thoracic vertebral morphological study investigating the four vertebral dimensions in Table 1, where measurements were taken from a population of either patients or cadavers.

**Table 2:** Range of vertebrae dimensions from previous morphological studies that analysed the thoracic spine in adults (Demiroz and Erdem 2020; Zindrick et al. 1987; Ebraheim et al. 1997; Tan et al. 2004; Lien et al. 2007; Pai et al. 2010)

	Range from study	Range of means from the literature	Standard deviation of minimum mean	Standard deviation of maximum mean
Pedicle Width (mm)	4.7–6.2	3.4–7.0	0.6	1.44
Pedicle Height (mm)	9.0–11.5	8.7–12.2	0.1	0.50
Transverse Angle (°)	3.5–12.5	5.9–25.9	0.9	3.3
Sagittal Angle (°)	13.5–21.2	4.0–27.3	0.8	1.5

Measurements encompass all three vertebral levels (T4–T6)

## Discussion

The measurements of pedicle dimensions in the first three PCs (Table 1) are within the range of means (Table 2) found in morphological studies, except the transverse angle, which showed a minimum of 3.5° in the SSM, 2.4° lower than the lowest mean in the literature (Tan et al. 2004).

The meta-analysis revealed much larger variations in vertebral morphology between different populations. Variations in results have also been attributed to differences in ethnic groups (Stockton et al. 2019) and sample sizes (Tan et al. 2004). The population used for training may not capture these distinctions. Additionally, the mean age of the patients in this study was 52.20 ± 9.16, and vertebral anatomy has been established to change with age (Holcombe et al. 2017). Therefore, caution must be used when assessing different demographics.

The studies in the literature took key measurements of each sample manually, either physically or with image analysis software. The SSM offers various advantages over this method. The morphology over many samples (270) was analysed using only 19 models (six standard deviation models for each of the three PCs + one mean model), reducing the amount of time needed for measurements. Also, the distance maps offer straightforward representations of variations in morphology that do not have to be assigned to a dimension and show correlations between features.

## Standardised surgical guides

A set of standard surgical guides would require a consistent anchor point where the variance of the shape of the vertebrae within the population is low. A well-established contact region is important as it will affect the alignment of the entire surgical guide and hence the resulting trajectory of the pedicle screw. The PCs seen in Fig. 5 show variance of up to 4 mm within the transverse process and spinous process, making these locations unsuitable as anchor points. However, the lamina shows variance of less than 1 mm in all PCs. This may make this a more suitable contact region for alignment of the guide. Despite this, the areas of low variance are very close to the screw entry points, making the design of a standardised set of guides challenging. It may be more suitable to design a guide that aligns using the lamina and then has adjustable aspects to anchor onto the transverse and spinous process, to prevent movement during pedicle screw insertion.

The results in Table 1 show the minimum range of trajectories and pedicle screw diameters that a set of standard guides would need to cover for a Korean population. For screw insertion without a breach, the authors recommend that the standardised guides accommodate pedicle screw diameters of 3.5–6 mm. There are numerous criteria for pedicle screw diameter selection (Solitro et al. 2019). However, screws smaller than 3.5 mm are seldom selected (and manufactured) and 6-mm screws are the largest screws that may be inserted into the largest pedicles without breach when considering the maximum pedicle width of 6.2 mm (Table 1).

With regard to the transverse pedicle angle, the authors recommend that the guides cover the range of 3.5°–12.4° (Table 1). However, unlike with the pedicle screw diameter selection, which is usually manufactured in defined increments of 0.5 mm, more work is needed to determine the transverse angle increment for which a set of standardised guides should be designed. If only considering the straightforward trajectory (where pedicle screw trajectory is parallel to the superior endplate), then the sagittal angle need not be considered, as the sagittal angle trajectory would be constant at 0°. This may be beneficial as it will allow a margin of error for the alignment process of the guide.

It is possible for there to be a combination of PCs that would result in a screw diameter or transverse angle that lies outside of the ranges identified above. When creating a model that was plus three standard deviations from the mean in PCs 1 and 3, and minus three standard deviations from the mean in PC2 (and inversely as well), the minima and maxima identified in transverse pedicle angle, width, height, and sagittal pedicle angle are outside the range quoted above. This, however, would likely only affect a very small number of patients. When creating a model of the same combination that was two standard deviations from the mean, the only dimension outside of the ranges identified was the pedicle height. Considering that pedicle height is the least important dimension (as screw diameter is always constrained by pedicle width) and that two standard deviations from the mean still cover approximately 95% of the population, a standardised set of surgical guides utilising the ranges identified above would still cover a wide range of the Korean population.

### Other statistical shape models of the thoracic spine

In terms of standalone presentations of SSMs, whilst there have been multiple SSM studies investigating the vertebrae in the lumbar and cervical regions (Clogenson et al. 2014; Hollenbeck et al. 2018), only one was found studying the thoracic vertebrae (Meakin et al. 2019). The SSM created by Meakin et al. (2019) accounted for 70% of the shape variation of 296 samples with the first five PCs. This is considerably higher than the variations found in this study (39.7% of variation in 270 samples with the first three PCs, Fig. 5). However, the SSM developed by Meakin et al. (2019) manually assigned landmark points for PCA meaning only 77 landmark points were chosen to represent each vertebra. This is much lower than the SSM presented above, where the number of landmark points in each vertebra was in the order of 2000. A greater number of landmark points give a better representation of the overall shape of the model. Therefore, capturing lower variance in the leading PCs is expected. Additionally, projection-pursuit PCA (ppPCA) algorithm used results in the leading PCs representing a lower proportion of the overall variance compared with classical PCA. The ppPCA algorithm is still more desirable due to its robustness in the presence of outliers (Croux et al. 2007). Furthermore, the SSM created by Meakin et al. (2019) included the entire thoracic region. Anatomy of the thoracic vertebrae varies significantly when not assessing directly adjacent levels, which is reflected in the large proportion of the variance in the leading PCs of the model.

### Other applications

This SSM may also be of use for other applications (Ambellan et al. 2019). Medical diagnostics and treatment are often based on an understanding on what “normal” anatomy is. However, due to variations within a population it can be difficult to diagnose and treat certain pathologies. A statistical representation of anatomy can therefore be very useful for computational diagnosis and therapy planning. Anatomy of the vertebrae may be missing or irregular due to fracture, pathological morphologies such as scoliosis, or even previous surgeries. This SSM may be used to generate the ideal shape for missing anatomy and aid spinal reconstruction surgery. For this, regularly shaped regions of the vertebra can be selected, with the aim of preserving this anatomy as best as possible. A shape can be generated using a linear combination of the PCs from the SSM. An ideal anatomy can then be produced by minimising the root mean squared surface distance between the generated shape and actual anatomy of these regular regions. Zachow et al. (2005) demonstrated this technique for the reconstruction of mandibular dysplasia.

The SSM could also be used as a training tool for students, allowing them to learn about the natural variations of the complete shape of the thoracic vertebrae within a population, instead of using a classical anatomical atlas containing discrete metrics. Though the population analysed in this study is relatively homogeneous, the statistical representation of anatomical variations may encourage students to develop a more intuitive perception of anatomy than current methods support.

### Limitations

This SSM grouped three vertebral levels (T4–T6) to produce results that could be used for all three levels. However, even between directly adjacent levels, anatomy of the vertebrae can differ slightly (La Barbera 2018). Therefore, if a surgical guide/algorithm specific to the vertebral levels is required, then three new SSMs would need to be produced. Mirzaalian et al. (2013) used a similar method to train the SSM, which resulted in acceptable levels of error, giving confidence to this method, albeit with the entire thoracic spine.

It is possible that the subsequent PCs that were not analysed further (with < 5% of morphological variation) could have implications for the proposed surgical guides. A further study designing and testing the surgical guides may wish to consider analysing more PCs, should the performance of the initial guides not be satisfactory. A further study must also appreciate that this SSM has been produced from a Korean population and may not be representative of a global population. Whilst the low root mean squared error implies that the SSM is representative of the Korean dataset, transferability to other demographics would need to be tested. This includes any effects of the ages and heights of the modelled population. Further studies could also empirically estimate what proportion of the Korean population the guide would cover. Additionally, as mentioned in Sect. 4.1 the PCs may be combined to produce models with dimensions outside of the ranges stated in this study. Exploring the various permutations of different PCs more thoroughly to identify a range of dimensions suitable for a wider range of the population may be of value.

Patient-specific surgical guides are often used when the anatomy of the patient is unusual, such as within individuals with scoliosis. In this scenario, this SSM may be unsuitable if the patient’s anatomy differs from “normal” anatomy a great deal. This study focused on the region where the pedicles are smallest and surgical guides are often used, even without abnormal anatomy.

## Conclusions

A statistical shape modelling pipeline was used to analyse the morphological variations of the T4–T6 vertebrae in a population of 90 adults (50 females and 40 males). Initial analysis of the first three PCs showed that a set of standardised surgical guides would ideally be aligned using the lamina of the vertebra due to the lower variance within the population at these locations. The guides would need to accommodate pedicle screw diameters of 3.5–6 mm and transverse pedicle screw angles of 3.5°–12.4°.
